# Oral Hygiene in Children with Autism: Teaching Self-Toothbrushing via Behavioural Intervention Including Parents

**DOI:** 10.3390/children12010005

**Published:** 2024-12-24

**Authors:** Marco Esposito, Carlotta Piersanti, Roberta Fadda, Marco Boitani, Monica Mazza, Giuseppina Marrocco

**Affiliations:** 1 Autism Research and Treatment Centre Una Breccia Nel Muro, 00168 Rome, Italy; 2 Department of Applied Clinical Sciences and Biotechnology, University of L’Aquila, 67100 L’Aquila, Italy; monica.mazza@univaq.it; 3 Department of Medical-Surgical Sciences and Biotechnologies, University of Rome La Sapienza, 04100 Latina, Italy; piersanti.1849068@studenti.uniroma1.it (C.P.); giuseppina.marrocco@uniroma1.it (G.M.); 4 Department of Pedagogy, Psychology and Philosophy, University of Cagliari, 09100 Cagliari, Italy; robfadda@unica.it; 5 Association of Social Promotion “Frammenti”, 04015 Priverno, Italy

**Keywords:** oral hygiene, tooth-brushing, autism spectrum disorder, applied behavior analysis, task analysis, video modeling, PECS, parent inclusion

## Abstract

Background/Objectives: Children on the autism spectrum experience more oral hygiene issues than peers, and tooth-brushing behavior seems particularly challenging for them since it includes diverse skills and collaboration. In this study, the efficacy is explored of a behavioral intervention mediated by staff and parents in teaching self-brushing teeth in eight autistic children. First, we wanted to examine whether the intervention improved self-brushing teeth skills in a short-term period. Second, we evaluated the long-term outcomes of the intervention. Finally, we analyzed the individual differences which might predict better outcomes. Methods: The training started during an ABA summer school with a supervised behavioral staff and lasted for eight sessions. The training package included several behavioral procedures such as prompting, fading, task analysis, chaining, differential reinforcement, direct instructions, visual aids, pictograms, and video modeling. According to a pre-and post-test design, we measured the frequency of independent self-brushing behaviors and interviewed the parents about the hygiene routines. Results: The results indicate a significant improvement in children’s self-brushing teeth behavior and maintenance, where 33.7% of the steps were achieved by children at baseline and 77.5% at post-training, and with four children, 100%. The parent questionnaires reported significant improvement in autonomy of self-brushing and times a day dedicated to oral hygiene. The severity of symptoms, sensory hypersensitivity, and lower IQ levels of the children negatively correlated with the outcome. Conclusions: These results point to relevant practical suggestions for families and clinical staff to address oral hygiene in the autism population.

## 1. Introduction

Children with autism spectrum disorders (ASDs) show social communication deficits and a tendency to engage in a pattern of restricted and repetitive behaviors commonly associated with comorbidities such as language disorders, hyperactivity, anxiety, challenging behaviors, and sensory anomalies [1]. All these symptoms and comorbidities might impact the general health and personal autonomies of children with ASDs, also depending on the symptoms’ severity [2]. Concerning the literature associated with the autonomies of children with ASDs, some researchers have investigated the oral status of this clinical population. Firstly, a systematic review examined oral hygiene in children and young adults with ASDs, showing more scarce hygiene in people with special needs than in the general population, particularly in the case of autism [3]. These studies reported 60.6% of caries and 69.4% of periodontitis, while risk factors included unhealthy diet, motor impairments, ineffective caregiving, hypersensitivity/fear, and some medications. Successively, other authors have highlighted low compliance in such a sample as a barrier to care, indicating a necessary collaboration between educational and medical professionals in increasing both a balanced diet and appropriate daily oral hygiene, reducing interventions via anesthesia [4]. In 2020, a meta-analysis of single case studies recommended a series of well-established indexes to assess oral hygiene, such as the prevalence of caries, periodontists, plaque, and gingivitis [5], which researchers should consider during their studies. Concerning challenging behaviors, another review confirmed a higher incidence of interventions in anesthesia than controls in children with ASD, shedding light on the lack of effective protocols to face the scarce collaboration of children during medical visits [6]. According to such evidence, a research group indicated a list of best practices in oral hygiene for people with special needs, such as an early diagnosis, preventive and interventional training, and adequate educational practices [7]. However, factors like financial costs, hypersensitivity, waiting time, scarce compliance, and the scarcity of behavioral teaching strategies limit such expectations [8].

In 2011, a letter on applied behavior analysis (ABA) and dental care argued that simple procedures applied by professionals during visits like tell–show–do are inadequate for patients with ASDs and behavioral issues. On the other hand, the provision of ABA can improve compliance during the visits and decrease the need for restraint and sedation [9]. Similarly, some authors explored the effectiveness of behavioral skills training (BST) in a group of dental hygienists who provided oral hygiene procedures to children with autism who exhibited challenging behaviors [10]. The treatment package consisted of instruction, modeling, role play, and feedback, including assessments of preferences, functional analysis, the Picture Exchange Communication System (PECS), discrete Trial training (DTT), incidental teaching, and guidance. In further interventions, such techniques also included differential reinforcement, visual schedules, in vivo and video modeling, reinforcement, sensory modulation, auditory cues, and narratives [11,12]. In a recent review of the four categories of oral hygiene management, orthodontist visits, treating challenging behaviors, and training professionals, people with ASDs received the following training: prompting/fading, escape extinction, modeling and video modeling, systematic desensitization, oral education, tangible/edible and social reinforcement, also with the token economy, PECS, BST, task analysis, and discrimination training [13].

In particular, visual aids have been largely applied in special education to increase the adaptive skills of children with ASD, such as social communication, comprehension, and autonomy [14]. Diverse studies have properly applied such strategies to increase oral hygiene. Firstly, a study that included 52 children with ASDs (3–19 years) examined an iPad application for oral hygiene that allowed the task analysis to convert sub-steps in images (16 created for children over six years and 12 for children under six). Children received 8 months of training once a week from a dentist who taught the sequential procedure through pictograms representing teeth and toothbrushing, involving their caregivers. Data scores ranged from 1 (absence) to 5 (independently), gradually fading the prompts. At baseline, no participant brushed their teeth, while ten children achieved this autonomy at the end of this study, still showing difficulties in brushing the dental surfaces since the occlusal result was easier than the buccal or lingual ones. Finally, many children continued to fail to spit [15]. Subsequently, thirty nonverbal children with ASDs (6–11 years) were involved in a study in Bulgaria [16]. The study lasted 12 months and was based on a non-verbal communication system since the images were in a sequence of actions for dental brushing. The study took place at home and comprised parent training following the Tell Show Do and Just Like Me protocols, helping family members by providing functional physical guidance in the most difficult passages and social reinforcements (praise). Successively, the families tried to fade the physical, verbal, and visual prompts for teaching the use of dental floss; the objective was that each participant, at the end of this study, could complete all oral hygiene actions in response to the simple instruction, brush your teeth. At the beginning of this study, 93% of the children had poor oral hygiene, half of the group brushed their teeth for less than a minute, and only 10% brushed their teeth twice a day. A significant improvement occurred starting from the third month of this study, while difficulties emerged in understanding verbal instructions, awareness of the importance of correct oral hygiene, sustained attention, coordination of the upper limbs, and tactile and oral sensitivity. The authors conducted a similar study for 30 blind children (7–14 years) without cognitive impairment who attended the Braille School [17]. Staff furnished some models of teeth with and without plaque/carious with Braille instructions about the correct movements to do with the toothbrush, accompanied by a melody, interrupted every time the child had to change position. The first year of exercise included training from a supervised dentist who guided each child, where the Tell Show Do technique changed to Tell Show Feel Do and training caregivers for generalization at home. From the first to third months of the program, they observed a significant improvement in oral hygiene. Another study underlined the efficacy of the PECS system in oral hygiene care via a toothbrushing program for 37 children with ASDs (4–16 years) [18]. All child–parent couples received two sets of images, where the first consisted of 10 small pictures to hang next to the bathroom sink at home, while the second was larger to include written instructions. The images showed the brushing sequence of all dental, occlusal, buccal, and lingual surfaces on the right and left sides for the maxillary and mandibular arches. Successively, parents received training on stages of PECS, while oral exams included analysis of plaque and gingival indexes. After the training, the gingival index significantly decreased, regardless of the age of the children, conversely for the plaque index. A limitation of the study highlighted that 75.7% of parents considered PECS hard to use but useful. Another study involved 60 adolescents with ASDs (13–17 years) to compare the effectiveness of paper-based visual tools with an interactive mobile application [19]. The inclusion criteria were CARS between 30 and 37, an understanding of visual and simple verbal instructions, and a plaque score between 2 and 3. The participants formed two groups in a non-randomized manner. The first group received 11 actual images illustrating the steps of teeth brushing, and the second followed a mobile application provisioned with an interactive game in which a character modelled the behavior offering reinforcement (each correct answer corresponded to one or more stars necessary for purchasing items in the application shop). Successively, children and their parents received modeling and had to brush their teeth at least two times a day for at least three minutes. A questionnaire collected information on oral health and three oral exams of plaque and gingival indexes at the beginning, after six, and after twelve weeks. The results showed a reduction in the plaque and gingival indexes in both groups, along with a general improvement in brushing habits and the caregiver’s perception of their children’s oral health status.

As shown, a video recording presents a subject who performs the action expected by the student. In a standard manner, the tutorial shows a person who models the target behavior, while in video-self-modeling, the tutor records the child during the same action, and in the children’s point-of-view, the child can observe his hands executing activities. Tutors can extract multiple clips or images from the video for a step-by-step explanation. The advantages of such practices are diverse, such as their use in different settings, accessibility, availability, appropriateness for diverse users, and cost-effectiveness [20]. Likewise, video-based interventions seem suitable for students with ASDs since they display a higher ability to process visual stimuli than vocal and visual over auditory information [21], increasing the attention toward relevant stimuli associated with the task [22,23]. Also, the teacher can reuse a video with other students or in a group and repeat previous trials. Generally, this practice requires minimal staff training on instructional delivery, and data can be personalized [24,25,26]. A quasi-randomized study compared two educational interventions on tooth brushing, social stories (narratives), and video modeling through a parent-mediated intervention [27]. The study enrolled 133 middle–high-functioning participants with ASDs in four schools (7–15 years) able to access the Internet and WhatsApp. At the beginning of the program, all children underwent an oral examination, which proved to be similar between the two groups. The parents and teachers of the intervention group participated in a meeting to receive information about the effects of sugary foods on tooth surfaces, the importance of plaque control, and the 3 min brushing technique to perform in school and at home; the tool consisted of a video where a ten-year-old child slowly showed the brushing phases in association with a background voice that described the steps delivering simple instructions. The control group received a different training without a live meeting and through brochures, along with a social story containing images and instructions on the 23 steps of the brushing technique. Successively, some reminders were sent to parents via WhatsApp three times a week to encourage the implementation of tools when brushing their teeth. Through a questionnaire to caregivers at the beginning and after a month, it was possible to identify a growing trend regarding parental knowledge and attitude towards their children’s oral health. The oral hygiene habits of the participants improved, while the level of consumption of sweet snacks remained unchanged in both groups.

The current study aimed to confirm the efficacy of behavioral practices in teaching self-brushing teeth in eight children with ASDs with parent inclusion, addressing the following research questions: (1) Did behavioral intervention improve oral hygiene and self-tooth-brushing of children with ASD? What were the demanding behaviors of task analysis to perform and acquire? (2) Did parents maintain levels acquired by children after the training? (3) Did higher cognitive skill levels and lower sensory anomalies of children at baseline represent an advantage in training outcomes?

## 2. Materials and Methods

The sample included eight children with ASDs (5.4–10.3 years; average = 8.1) enrolled in a treatment center where they had been receiving behavioral intervention [28]. The clinical staff enrolled the participants in an ABA summer school. Therefore, the supervisor selected a purposive sample to participate in this study since she had collected data from their previous interventions and guided the research design and aim of the training. The inclusion criteria of the sample included the following: diagnosis of an ASDs performed by an external neuropsychiatric with ADOS-2, Raven’s Matrices, and no medical/genetic impairments.

Case A is a female able to emit a few vocal requests with tactile and oral hypersensitivity, refusing both tasting adverse stimuli and mouth manipulations (IQ = 75–85; ADOS level 3). She displayed screaming and crying 7–10 times a day with the escape function. She did not comprehend the vocal instructions and benefited from the visual support. At the beginning of the training, she had difficulty accepting the toothbrush and toothpaste inside the oral cavity and refused any form of lateral brushing.

Case B is a female, non-verbal communicating with PECS (IQ = 45–50; ADOS level 3), showing hypo-sensitivity and absence of severe challenging behaviors. At the beginning of the training, she had not completed any steps independently.

Case C is a male, non-verbal subject communicating with PECS (IQ = 65–75; ADOS level 3). The child displayed severe food selectivity and oral hypersensitivity. The problem behaviors had the function of escaping from the tasks, not activated during oral hygiene times. At baseline, he needed a hand–hand prompt.

Case D is a male, and his vocal communication included expressions for request purposes (IQ = 85–95; ADOS level 3). He displayed problem behaviors of hetero-aggression contingent on some instructions. During the oral hygiene procedure, no aggression occurred. He showed hyper-sensitivity. During training, he followed the PECS along with physical prompts.

Case E is the youngest male with hyperactivity. His vocal communication included requests, comments, and descriptions (IQ = 95–105; ADOS level 1). He displayed tactile, oral, tactile, visual, and movement hypersensitivity. At baseline, the child performed some oral hygiene steps independently. He exhibited challenging behaviors contingent on instructions but not during oral hygiene.

Case F is a male with non-verbal communication and hyperactivity using PECS (IQ = 75–85; ADOS level 3). Problem behaviors occurred with escape functions from the tasks. At baseline, the child showed some autonomy in the preparation phases of brushing.

Case G is male, with communication including vocal requests and comments (IQ = 95–105; ADOS level 2) with oral hypersensitivity. Challenging behaviors occurred when educational activities started (escape). At baseline, the child demonstrated some abilities regarding oral hygiene.

Case H is male, using PECS, showing scarce collaboration during IQ assessment by displaying poor attention and severe self-stimulating (ADOS level 3). Also, he showed problem behaviors to escape, throwing away the items provided.

This study followed BACB’s ethics requirements (https://www.bacb.com/ethics-information/ethics-codes/, accessed on 14 February 2023) since the supervisor informed parents of participants about the nature of the research and significant factors that could influence their willingness to participate; answered any other questions participants had about the research; and informed them that they were free to depart from the research at any time without penalty. The research group received written consent from parents and authorization from the local health ethical committee (Protocol n.19233 of ASL Latina).

Firstly, we selected indirect and direct measures to gather the following information: symptom severity, cognitive level, sensory profile, autonomy in personal hygiene, and self-brushing behavior. The selected Short Sensory Profile (SSP) [29] assessed the hyper- or hypo-sensitivity, including oral sensitivity. This questionnaire identifies problems in sensory processing in children on 38 items divided into 7 subscales: tactile sensitivity, taste/smell sensitivity, movement sensitivity, under responsiveness/seek sensation, auditory filtering, low energy/weak, and visual/auditory sensitivity. The mothers answered the questions on a five-point Likert scale, from never (for 0% of the time) to always (100% of the time). The responses calculated the score of each subscale to obtain a total score, which will be classified as typical if within 1 standard deviation (SD) from the reference mean, probable difference if it is between 1 and 2 SD, and definite difference if it is 2 SD below the mean. Low scores on each subscale indicate a greater sensitivity in the child, and some authors found that items discriminated against children with ASDs by TDC [30].

Also, we selected an interview with parents as the SVAP-R [31] to gather the hygiene routine levels of their children, containing seven scales, such as basic skills, cognitive skills, personal autonomy skills, socio-emotional skills, academic skills, integrative skills, and challenging behaviors, with items ranging from 0 to 3 based on the level of mastery, where 0 indicates that the child does not perform, score 1 corresponds to a prompted performance, score 2 corresponds to a skill emitted partially, and finally, score 3 indicates skill mastered; since the questionnaire was particularly extensive, we selected only the personal hygiene dimension via 12 items such as washing hands and face, other parts of the body, noticing if he is dirty, using the shower, brushing teeth, hair, reporting dirty or trimmed nails, combing hair, intimate hygiene, blowing nose, drying, and cleaning sink or tub.

Finally, we furnished an 8-item questionnaire on the oral hygiene routines of children to their parents on the following items: how many times a day does he brush his teeth? Does he use a toothbrush? Does he use dental floss? Does he use an interdental brush? Does he use a mouthwash? Does he take fluoride tablets/drops? When do you go to the dentist? How many times a day do you consume sugary foods or drinks? (responses included 0 = never; 1 = sometimes; 2 = always).

Regarding direct measurement, we registered the frequency of correct or prompted behaviors on self-brushing teeth, dividing the behavior into twelve steps (task analysis) to teach the behavioral chaining in a natural form such as opening the tap water, taking the toothbrush, putting the toothbrush under water, putting toothpaste on the toothbrush, brushing teeth on top, down, left, and right, cleaning the mouth, spitting, closing the tap water, and finally drying hands/mouth. The performances on each step scored 0 in the case of no response, 1 in the case of prompted response, and 2 for unprompted response.

Since the sample was limited and goal-directed, we studied each participant via an ABA-C single case design (SCD) with a month of follow-up where the effects of an intervention (B) are evaluated by alternating the baseline condition (A phase), and at the end of a month follow-up (C) [32]. The initial phase starts with baseline observations when behavior is observed under conditions before intervention is employed. During baseline, the behavior was unprompted, and therapists merely asked participants to brush their teeth, collecting the responses. The baseline continued until the rate of the behavior appeared to be stable (2 data points). The entire training included three sessions about baselines, eight training sessions, and a follow-up.

### 2.1. Training

The clinical staff comprised three behavioral therapists who provided one-to-one training and a behavioral analyst who provided individual supervision for 20% of the training. Firstly, before implementing training for oral hygiene, children received a one-to-one brief stimulus preference assessment (SPA) in free operants to detect the stimuli as reinforcers [33]. The reinforcers were chosen in a range of activities on a Likert scale from 0 (no preference) to 5 (maximum preference). Generally, the reinforcers were external to the oral hygiene procedure. Successively, the behavioral staff provided some stimulus–stimulus pairing sessions to increase the collaboration and compliance of children with materials, settings, and people [34]. All parents participated in two parent-group training meetings where the staff described the behavioral procedures. The families were involved in completing the questionnaires both before and after the training. During summer school, after lunch, each child went to the bathroom to brush their teeth for eight sessions. The setting included a school bathroom with three sinks where two tutors were assigned randomly to each child. The behavioral package included prompting/fading, task analysis/chaining, differential reinforcement, direct instructions, visual aids, PECS, in vivo, and video modeling. The children following the picture schedules had to follow a visual sequence of 12 steps. As a prerequisite, they had to match an action card with a self-actual movement before following the chaining [35]. Initially, only six of eight children (A, B, D, E, F, and G) demonstrated imitation of a single card or a sequence of cards (PECS), of whom the last three also imitated a video sequence (video modeling).

The training started with initial stimulus discrimination (SD), asking the child to brush his teeth and pointing to the first picture as a prompt (opening the tap water). If the child did not show the correct behavior, then the staff employed more invasive prompts, such as physical guidance and verbal prompts [36]. The correct behaviors received a reinforcement with a preferred stimulus previously selected. Successively, the therapist faded the prompts as the child displayed autonomy in performing the task, applying differential reinforcement (DR) [37]. The behavioral chaining followed a natural process via a total task technique since the therapist prompted and reinforced the target behaviors until the end of the chaining [38]. The master criterion of the task was 100% unprompted response for only one session, where the therapist would have applied a variable reinforcement schedule rather than a continued one [39]. The video modeling included 12-step point-of-view video modeling through a smartphone following established guidelines [40,41,42]. The children watched the first video, showing the hands of a person who performed the steps to follow. Also, the children could pause and rewatch the tutorial to self-manage the task [43]. If the child needed a more invasive prompt, it was implemented and faded after applying a DR schedule. The master criterion was the same as the previous condition, and therapists varied reinforcers to increase their value or transferred them into a token economy strategy [44]. All the therapies followed a one-to-one ratio between children and therapists and 20% of indirect and direct supervision. Also, the case manager provided individual parent training to caregivers (one hour a week) to transfer and generalize the educational method at home [45]. At the follow-up, the caregivers reported to the staff the following information: if the child brushed teeth autonomously, how many times a day, and if the child passed to an electronic toothbrush.

### 2.2. Data Analysis

We collected indirect and direct measures such as symptom severity, cognitive level, sensory profile, personal hygiene, and self-brushing behavior. In the first hypothesis, we endeavored to monitor training in improving self-brushing and oral hygiene. Regarding the direct measurement, the dependent variable was the percentage of mastered steps of task analysis of brushing teeth in every data point (three repeated baselines, eight data points of sessions regarding training, and 1-month follow-up). For example, when the staff scored 2 for an unprompted response to a single step of task analysis, then we calculated the sum of all numbers 2/12 steps of chaining*100 for every data point, plotting eight dispersion graphs. The percentage of steps carried out independently was calculated pre- and post-training, individually and for the entire group, so individual scores were analyzed in SCD, and the group served to study associations with precursors such as the severity of symptoms, IQ, and hyper/hypo-sensitivity.

Moreover, to highlight which steps of chaining resulted in more difficulty than others to acquire from participants, we reduced the 12 steps into 5 clusters via k-means algorithm (explained variance ratio: 0.95) as follows (1–2, 3–4, 5–6, 7–8, 9–12), SSE (within: 6.35), SSG (between groups: 125.64), SST (total: 132). In such a manner, we could plot a dispersion graph related to clusters, highlighting pre- and post-training on which segment of chaining children failed.

Also, SVAP-R interviews on oral hygiene included ordinal scores treated with non-parametric tests. Subsequently, we endeavored to compare children pre- and post-training, considering that data were dependent. As a result, we established 2 × 2 classification tables for each item. For example, the first item stated he washes his hands and face, then the participants were classified into the following four categories: positive/positive (how many children wash hands both at pre- and post-training?), positive/negative (how many children did miss the previously acquired item?), negative/positive (how many children did improve washing hands only at post-training?), and both negative (children failed both at pre- and post-training). We applied pre- and post-non-parametric tests (McNemar) on each classification table for the entire group.

Finally, the third hypothesis regarded the impact of precursors such as IQ level, sensitivity, and autism severity. The Raven’s matrices and seven sensory profiles provided metric variables, except the qualitative variable of severity level (from 1 to 3) and the dependent variable being frequencies. As a result, we calculated a Spearman correlation between these variables for the entire group. We performed tests with alpha <0.05. Data remain available via request on writing to the corresponding author.

## 3. Results

Firstly, we describe the individual scores of children on both direct and indirect measures collected (the process of task analysis acquisition, scores on sensory scales, and reports on oral and personal hygiene).

Case A. At baseline, she displayed 30% of acquisition, while 100% at post-training, with discrete follow-up maintenance (80%). The parents reported fatigue and often assisted the child in brushing with physical guidance. At baseline, the child brushed her teeth frequently once a day, while at follow-up, she increased to more than once a day, using only toothbrushes and toothpaste. In the SVAP questionnaire, a discrete improvement emerges in other autonomies of personal hygiene. Finally, the sensory scales that surpassed 2 SD from the reference mean were tactile, taste/smell, and auditory filtering scales.

Case B. At baseline, she did not display step acquisition. At post-training, she learned to manage the toothpaste, open and close the water tap, partially squeeze out the toothpaste, and brush her teeth (70%). The SVAP showed a discrete increase in other hygiene routines. Concerning sensory anomalies, she showed 2 SD from the reference mean under responsiveness/seek sensation, auditory filtering, and visual/auditory scales. Another change was the replacement of the manual toothbrush with an electric one and the inclusion of mouthwash. Finally, she increased the frequency of brushing her teeth to twice a day.

Case C. At baseline, he did not display acquisition, while at post-training 40% of steps. He needed a hand–hand prompt progressively reduced. At half training, the child accepted the toothbrush into the oral cavity by brushing the teeth for the four arches or at the upper and lower incisors. Also, he showed severe impairment (2 SD) in all sensory scales. In the SVAP questionnaire, a slight increase in personal hygiene occurred. Concerning parent inclusion, they prompted the child two times a day using an electric toothbrush. At follow-up, an increase in the frequency of brushing teeth was observed from 1 time to 3 times a day.

Case D. At baseline, he displayed 30% acquisition, while at post-training 100% and at follow-up. Even if he displayed challenging behaviors in other circumstances, no aggressive behavior occurred during the training. In the initial phase, he received a guide by the images and physical prompts and successively demonstrated ability to proceed independently. He showed sensitivity (2 SD) in the three sensory scales of under responsiveness/seek sensation, auditory filtering, and visual/auditory. The child followed the hygiene procedures only with the father, using an electric toothbrush and passing from 1 to 3 times a day to brush his teeth.

Case E. At baseline, he displayed 50% step acquisition, while reaching almost the full achievement at post-training and follow-up. At the end of the training, he self-brushed his teeth with partial prompts. He showed sensitivity (2SD) in the three sensory scales of taste/smell sensitivity, under responsiveness/seek sensation, and auditory filtering. The child replaced the manual toothbrush with an electric one, increasing the frequency of brushing from 1 to 3 times.

Case F. At baseline, he displayed 80% step acquisition, while at post-training 100% and at follow-up. At baseline, the child showed autonomy in the preparation phases of brushing, so the training contributed to inserting more physical strength into brushing and spitting out water from the mouth. At the follow-up, the child utilized the electric toothbrush using a mouthwash. The daily frequency of his toothbrushing passed from 1 to 3 times a day. He reported only 2 SD from the reference mean on the taste/smell sensitivity scale.

Case G. At baseline, he displayed 80% acquisition, while at post-training 100% and at follow-up. At baseline, the child demonstrated the ability to carry out the sequences of screwing, pushing the toothpaste inserting it into the toothbrush. Through the video modeling, the child learned to brush his teeth independently. He reported 2 SD from the reference mean on diverse sensory scales such as taste/smell sensitivity scale, movement sensitivity, auditory filtering, and visual/auditory sensitivity.

**Figure 1:**
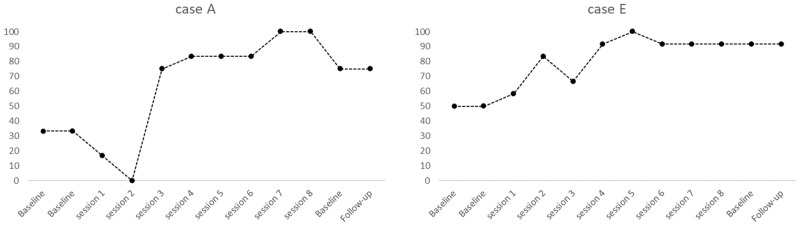

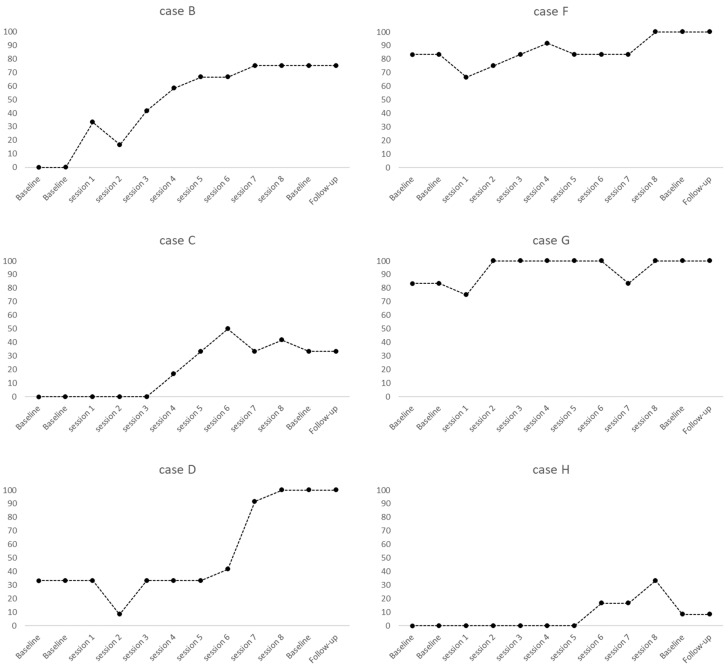
Individual task analysis. Percentages of mastered 12 steps during baselines, training sessions, and 1-month follow-up. Note. A, B, and D followed a behavioral package including pictograms, then E, F, and G used video modeling, while C and H received mainly physical prompts. Participants were less heterogeneous at post-training than at first baselines.

Case H. At baseline, he did not display step acquisition, while at post-training 30%. He displayed escape and sensory stimulation, throwing away objects. During the training, the child remained dependent on the therapist’s prompting. Also, he reported severe impairments in almost all sensory scales. The SVAP revealed a slight improvement in personal hygiene skills. Unfortunately, the child’s oral hygiene habits did not change over time, although the electric toothbrush replaced the manual one. It is possible to observe the skill acquisition for each participant in Figure 1.

Concerning questionnaires, families reported an increment in the frequency of diverse hygiene routines from their children, such as washing hands and other body parts and blowing noses. However, this evidence did not reach a significant difference pre- and post-training. On the other hand, the entire group were significantly and independently ameliorated for their parents in the self-brushing routine (Mc = 87.50, *p* = 0.015), doubling the frequency of self-brushing a day (Table 1). Also, we did not count the difference when children passed from a manual toothbrush to an electric one. The supervisor suggested this change when searching to reduce some barriers to the current intervention.

**Figure 2:**
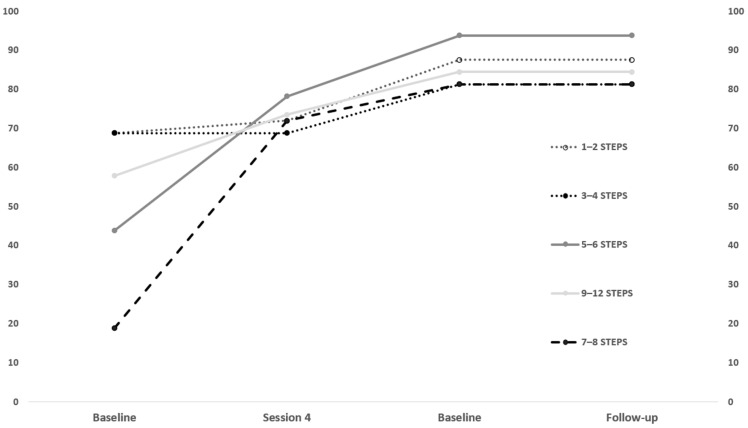
Difference in step acquisition of the task analysis. Note. We chose the fourth session as an additional data point to better visualize the process since it was intermediate.

Clinical staff focused on specific behaviors of task analysis that resulted in difficult achievements for children, requiring precise prerequisites related to the composite movements. We reduced the twelve steps of task analysis into five clusters (explained variance = 0.95) as follows: 1–2 = opening the tap water and taking the toothbrush; 3–4 = toothbrush under water, putting on toothpaste; 5–6: brushing teeth on top and down; 7–8: brushing teeth on left and right; 9–12: cleaning the mouth, spitting, closing the tap, and drying mouth. The performances scored 1 for a prompted response and 2 for independent until having a percentage of step acquisition on each data point (Figure 2). At baseline, since the training was in forward chaining (natural sequence), the first four steps (1–4) resulted in more achievement than other clusters, while the others increased during the training. At post-training and follow-up, some clusters, such as brushing teeth left and right (7–8) and spitting (9–12), resulted in more difficulty of achievement, while the steps more easily resulted in brushing teeth on the top and down. Such differences in step acquisition resulted in a significant difference (Chi^2^ = 31.088; *p* = 0.001).

### 3.1. Maintenance: Follow-Up and Generalization

Although only case D reached 100% success using pictograms, the other children started without acquisition, showing higher success in pre- and post-training ratio (A: 30–80%; B: 0–70%; C: 0–40%; D: 30–100%; E: 50–100%; F: 80–100%; G: 80–100%; H: 0–30%). The mean of the group at baseline was 33.7 versus 77.5 at post-training (SD = 33.7; 28.6), and the t-test for two dependent means displayed t = 6.201 with *p* = 0.000. The maintenance and generalization of self-brushing behavior showed a constant ratio between post-training and follow-up.

### 3.2. Precursors: Symptom Severity, Cognitive and Sensory Anomalies

Firstly, in comparison to level 1 (only two children out of the group) or level 3 of autism severity, there was a clear difference between the two starting points where low-functioning children achieved lower scores at the beginning of training and also at the end than high-functioning ones (level 1 gained on average 67% and 96% at pre- and post-training than 25% and 65% of level 3). Although the correlation showed a trend in such association the data did not achieve significance, while the IQ scores were significantly correlated with self-brushing at pre- and post-training since a higher IQ guaranteed higher acquisition in self-brushing (rs = 0.81 and 0.75, respectively; *p* ≤ 0.05). Additionally, there were relevant results from the analyses of the sensory profile scales. Specifically, the following taste/smell (rs = 0.72) and auditory filtering (rs = −0.70) were significantly correlated with self-brushing scores (*p* = <0.05). See Table 2 for further details on the correlation of precursors and outcomes.

## 4. Discussion

In the current study, the effects of behavioral intervention in self-toothbrushing were investigated in eight children with ASDs. Children in spectrum conditions could experience significantly more oral hygiene issues due to diverse risk factors [3,4,5]. About 60–70% of caries and gingival issues were discovered in these clinical samples, although some moderators could intervene, such as unhealthy diet, sensory anomalies, challenging behaviors, and caregiving [6]. Regarding the educational assistance furnished, an increase in compliance of children with ASDs occurred when professionals implemented an ABA intervention. Behavioral training reduced restraint and sedation during the visits, supporting professionals and parents to accomplish the procedures modelled [9,10,11,12]. Concerning the behavioral techniques successfully applied in these cases, the studies other than visual and video aids have included the following strategies such as prompting/fading and reinforcement, token economy, arrangement of environmental, differential reinforcement, narratives, task analysis/chaining, and parent training [13], showing some toothbrushing difficulties by children such as hygiene of the buccal and lingual surfaces other than the occlusal one, spitting, and use of pictograms by parents [18].

Regarding the first research question (1), we confirmed that children receiving behavioral training obtained an advantage in oral hygiene, either in improvement in frequencies of toothbrushing step acquisition or in parental reports. Also, children significantly increased their daily time for self-brushing their teeth, and some parents declared that their children received visits to dentists without sedation. Post-training, children showed similar improvement and a minor group variance, probably because the behavioral packages applied by tutors guaranteed specular training. The tutors worked daily with the ABA staff, receiving consultation from the same supervisor.

Concerning the research design, the participants followed an SCD with follow-up, showing acquisition in every data point on self-brushing teeth, and such methodologies permitted us to monitor rigorously the behavior acquisition, studying similarities and differences between participants. At the same time, other hygiene behaviors of children reported by their families (questionnaire) increased, such as washing hands, face, and other body parts, even if they did not reach significance. It is possible that parents felt confident enough to ask their children to complete other hygiene routines (not trained by therapists). Also, some steps were significantly more difficult for children than others, such as brushing teeth left and right, spitting, and the final steps of chaining. In particular, spitting could require speech training in buccal lingual praxis rather than practice during the chaining with repeated discrete trials. Likewise, brushing on the left/right resulted in more difficulty than the top/down movement, evidence frequently observed in other personal and extra-personal spatial competencies. The literature has suggested that some dentists address this problem by providing a denture to the child to offer practice and then transferring it successively to teeth.

The second research question (2) investigated the maintenance of achievements. At post-training and follow-up, we observed independence in step acquisition and maintenance in only four children, those of cases D, E, F, and G, than cases A (80%), B (70%), C (40%), and case H (30%). In some cases, we noted a discrepancy between the training furnished at summer school and parent-mediated practice, and probably staff observation could explain part of this failure. Concerning case A, her parents continued post-training to provide partial physical guidance, noting and declaring that her hypersensitivity (tactile, taste/smell, and auditory) was probably an obstacle to her success, along with frequent escape instructions. Case B exhibited sensory issues such as seeking sensation, auditory filtering, and visual/auditory, which could similarly affect his learning. Cases C and H started at baseline with a more severe profile than peers, lacking some prerequisite to learn or to replicate a visual procedure along with a scarce comprehension of prompting furnished by therapists. To respond to the inclusion of families, some aspects of training could be ameliorated by staff, guaranteeing a sufficient generalization. For example, staff could furnish more material to train families and educational support, as well as a reminder of times of procedure to follow at home. On the other hand, the experience of summer camp is limited. Staff have continued to support the same families after the training, and families have reported better results, even if we did not gather additional follow-ups.

Regarding the task analysis, the middle and final merged steps of chaining, such as brushing teeth left and right (7–8), cleaning the mouth, spitting, closing the tap water, and drying hands/mouth (9–12) showed a scarcer increment than initial steps of chaining, in line with the previous literature. Since some behaviors seem hard to learn in a quasi-natural process by children with ASD, therapists could require them in DTT, such as spitting in a bowl at the table to decrease attention load. As a result, after a single behavior reaches a masterization (stability of unprompted behaviors), it might be transferred into chaining. Also, staff could offer pre-training by dividing a single behavior into multiple steps or transferring a single step (brushing to left and right) into another play activity by brushing the mouth of a toy.

Finally, the third research question (3) asked if IQ and sensory anomalies correlate with outcomes. The correlations showed that low cognitive functioning was associated with fewer results. Children with cognitive impairment could lack diverse prerequisites to address the current training. For example, a child had to maintain attention for consecutive steps, replicate an action by picture, and imitate a tutor in a video. Ensuring such a prerequisite in a child could be necessary for including them in the training when achieving previous base skills. Hence, the current results indicated a higher beneficial effect in children with higher IQ pre-and post-training. These results are in line with previous studies, showing how cognitive and language development are relevant predictors of success for any intervention for children with ASD. Furthermore, executive functions associated with attention and chaining, including skills like planning, shifting, and working memory, could sustain self-care abilities, including toothbrushing. In conclusion, sustained/divided attention for children, moving from the previous step to the subsequent, should be examined in such a way as to program a compensative tool like a timer or a tailored agenda. Severe symptomology of spectrum conditions and cognitive impairment are well-known barriers to behavioral intervention. In these cases, clinical staff should increase the basic skills of their children using a functional assessment to furnish a tailored supervised program. Also, sensory anomalies such as tactile and auditory filtering correlated with outcomes, showing that precise sensitivity could affect learning. Children with severe sensory modulation could show less compliance and overreact in the presence of adverse sensory stimuli. Taste and smell sensitivity decreased post-training in children by increasing chaining acquisition. When a child displays severe sensory issues, therapists could offer pre-conditioning sessions to reduce the future barriers to toothbrushing training. Another aspect of such a condition concerns the environmental modifications (light, space, colors, objects, movements, and audibles) and tools employed during the training, such as manual, electric, or special toothbrushes, typology of toothpaste, mouthwash, and dental flosses. Finally, a profound clinical knowledge of the child can guide toward a beneficial change, such as often conditioning materials and activities.

The current study shows merit in its diverse advantages. Firstly, the staff measured the dependent variable, directly or indirectly (SCD and via a questionnaire), guaranteeing convergent validity. The target behavior included repeated measurements intra-between subjects to control internal validity. The families participated and followed their children at home. However, we have suggested additional changes in training since staff perceived the generalization process as unstable. Nevertheless, the parents participated before, during, and after the intervention, allowing the maintenance of the target behavior from the therapy settings to the natural environment. Also, parent inclusion increased the frequency of the exposure to the task, which might have led to a long-term positive effect of the training (also after the training as declared by the same families). Moreover, the results of this study have important implications for the quality of life of children with ASDs and their families. Learning how to improve oral hygiene might reduce possible invasive and stressful dental treatments, like ultrasonic teeth cleaning or cavity management, which might require complete sedation for children with ASDs. Also, reducing the frequency of dental visits means less stress for the children and their parents and less financial costs for the family. According to informal parental reports, it also seems that other personal hygiene behaviors improved after the training. Parents’ sensitivity to self-care skills related to the personal hygiene of their children can lead them to train the children in other self-care abilities like washing the hands, face, and other body parts.

Despite the promising results, our study has some limitations since the children showed heterogeneous characteristics (four rapid responders, two intermediates, and two severe) in terms of the severity of the disorder and cognitive level, as well as in terms of self-care and daily living abilities; more studies with more homogeneous and numerous samples would need to confirm our results; the current study did not involve the dentists since the program started from an ABA summer school, even if such collaboration might guarantee higher monitoring of oral hygiene via well-established caries and gingivitis indexes [46] and their involvement in the assessment and treatment [47]. Collaboration between local dentists and the supervisor is currently constant. Another issue of research design was the effort to evaluate the effect of the single components of the training package since it did not comprise a component analysis permitting the manipulation of a single component of a behavioral package [48] because the clinical staff did not manipulate pictograms, in vivo and video modeling as independent variables. Also, in this study, an interobserver agreement (IOA) was not included which could have guaranteed the fidelity of this study, and nor was integrity monitoring of procedures applied. Future studies are therefore needed to overcome the current limitations. Finally, a recent study confirms the significant challenges faced by parents of children with ASDs in managing their oral health, highlighting a worse perceived oral health-related quality of life compared to that of neurotypical peers [49]. The parents of a large group of children and adolescents with ASDs received oral hygiene instructions and dietary recommendations during the admission visit and follow-up visits. Also, children and adolescents with ASDs received regular oral hygiene sessions and dental care interventions. The study included a similar group of children and adolescents with ASDs who did not receive any intervention for oral hygiene and a larger control group of typically developing children and adolescents. The results indicated that oral hygiene is crucial for reducing parental stress and increasing the quality of life in children and adolescents with autism and their families. These results need to develop effective educational programs that might include mobile technology, such as video prompting and visual aids, to implement highly individualized interventions.

## 5. Conclusions

The current study offers a brief overview of the oral health issues in children and adolescents with autism spectrum and effective behavior practices to help them avoid restraint and sedation in case of challenging behaviors. The research included eight children with ASDs and their parents from an ABA summer school following behavioral training on self-tooth-brushing behavior, including such practices as task analysis, visual schedule, and video modeling, demonstrating the advantages of this treatment. The results showed advantages in children in acquiring oral hygiene skills and shed light on the best practices and arrangements to follow in natural and medical settings. Also, the parents of children reported other achievements of training concerning behaviors not trained, highlighting the importance of including families in the intervention. Moreover, in this study, an association was confirmed between symptomatology, cognitive abilities, and the sensory anomalies of children which affected their self-brushing abilities, suggesting consideration of these dimensions as a prerequisite to the training. Further research might include dentists guaranteeing children with challenging behaviors a safe and confidential setting during medical visits and reducing the use of restraints and sedation.

## Figures and Tables

**Table 1:** Parents’ reports on hygiene routines of their children pre- and post-training.

Personal Hygiene (SVAP)	Pre-Training	Post-Training	Diff.	95% CI	*p*
He washes his hands and face	4	8	50%	[15–84]	0.125
Washes other parts of the body	4	7	37%	[04–71]	0.250
He notices if he is dirty	4	6	25%	[-05–55]	0.50
Uses the shower	4	5	12%	[-10–35]	1.00
He brushes his teeth	0	7	87%	[65–100]	0.015 *
She washes her hair	0	2	25%	[-05–55]	0.50
Reports dirty or trimmed nails	0	2	25%	[-05–55]	0.50
He combs his hair	1	2	12%	[-10–35]	1.00
Takes care of intimate hygiene	2	4	25%	[-05–55]	0.50
He blows his nose	2	5	37%	[04–71]	0.250
Dries	4	6	25%	[-05–55]	0.50
Cleans sink or tub	1	2	12%	[-10–35]	1.00
**Oral Hygiene Questionnaire**	**Pre-training**	**Post-training**	**Diff.**		
Toothbrush routine	8	8			
Dental floss	0	0		Na	
Interdental brush	0	0			
Mouthwash	1	2	12%	[-10–35]	1.00
Fluoride tablets/drops	0	0		Na	
One time a year visit a dentist	4	6	25%	[-05–55]	0.50
One times × day consumes sweets	7	6	12%	[-10–35]	1.00
Two times × day brushing teeth	2	8	75%	[45–100]	0.031 *

Note. The scores are relative to how many children demonstrated the related behavior for their parents at that moment. * α < 0.05; Na = not applicable.

**Table 2:** Correlation analysis of precursors with self-brushing chaining.

	Pre-Training		Post-Training	
Variables	r	*p*	r	*p*
Symptom severity	−0.55	0.150	−0.35	0.390
IQ levels	0.811	0.014 *	0.750	0.032 *
Sensory scales (SSPs)				
Tactile Sensitivity	0.172	0.682	0.721	0.043 *
Taste/Smell Sensitivity	0.239	0.481	−051	0.187
Movement Sensitivity	−0.49	0.217	0.403	0.321
Under Resp./Seek Sensation	0.475	0.234	−0.48	0.227
Auditory Filtering	−0.70	0.049 *	0.080	0.849
Low Energy/Weak	0.024	0.954	−0.28	0.495
Visual/Auditory Sensitivity	0.093	0.826	−0.35	0.390

Note. Two-tails distribution with α < 0.05 *.

## Data Availability

All data gathered during the intervention are available. Please send an email to the corresponding author to access to database.
